# Face and content validity of the Carer Support Needs Assessment Tool (CSNAT), and feasibility of the CSNAT intervention, for carers of patients with chronic obstructive pulmonary disease

**DOI:** 10.1177/1742395321999433

**Published:** 2021-03-24

**Authors:** Kerry Micklewright, Morag Farquhar

**Affiliations:** School of Health Sciences, University of East Anglia, Norwich Research Park, Norwich, UK

**Keywords:** Chronic disease, caregivers, chronic obstructive pulmonary disease, focus groups, intervention

## Abstract

**Objectives:**

Informal carers of patients with Chronic Obstructive Pulmonary Disease (COPD) have unmet support needs. Evidence relating to carers’ support needs in chronic conditions informed version 3 of the Carer Support Needs Assessment Tool (CSNAT) which forms part of an intervention to identify and address carer support needs. Aim of study: to establish the face and content validity of CSNAT v3 for use with COPD carers and explore their views on delivery of the CSNAT Intervention in practice.

**Methods:**

Focus groups conducted September-October 2019 in non-clinical settings recruited eleven COPD carers (two to six participants per group). COPD patients (*n* = 2) attended one group to facilitate carer attendance, the impact of which is discussed. Most participating carers were female (*n* = 10); carers’ ages ranged 52–79 years.

**Results:**

CSNAT v3 was easy to understand and complete, and all 15 domains were considered relevant and appropriate, suggesting good face and content validity. The demeanour, relational skills, and knowledge of the CSNAT facilitator appeared more important to carers than being a certain practitioner type.

**Discussion:**

COPD carers considered the CSNAT Intervention an acceptable way of identifying and responding to their needs. The intervention could potentially be delivered through a range of services.

## Introduction

Informal carers are “lay people in a close supportive role who share in the illness experience of the patient and who undertake vital care work and emotion management.”^1^ Carers are often key in supporting patients with chronic or progressive conditions such as Chronic Obstructive Pulmonary Disease (COPD): an estimated 77% of people with advanced COPD have carers.^2^ This supportive role can impact on carers’ health and wellbeing; many have support needs that go unidentified or unaddressed. A recent review found areas of unmet need related to insufficient information provision, poor support to manage emotional distress, social isolation and access to services.^3^ Carers are also patients with their own direct support needs. Policy increasingly emphasizes the importance of carers in sustaining health and social care services whilst recognising the need to provide them with person-centred support.^4–7^

Health and social care practitioners are well-placed to enable identification and addressing of COPD carers’ support needs: the Carer Support Needs Assessment Tool (CSNAT) Intervention could facilitate this.^8^ The CSNAT Intervention consists of: (1) an evidence-based tool completed by the carer which is integrated into (2) ‘The CSNAT Approach’, a five-stage person-centred process of assessment and support that is practitioner facilitated but carer led.^9^ Following introduction (Stage 1) and completion of the tool (Stage 2), a needs-led conversation between carer and practitioner identifies and prioritises the carer’s unmet support needs using the carer’s self-completed tool (Stage 3). The carer and practitioner together then tailor responses to the prioritised needs (Stage 4); outcomes are reviewed at a later point (Stage 5) and the process repeated at an agreed time. Specific responses depend on the need identified, carer preference and available resources but may include active listening (validating carers’ expression of need), education, signposting or onward referral.

The CSNAT itself (the tool) was initially developed with carers of people predominantly with end-stage cancer: its relevance to COPD carers was relatively unknown.^10^ Micklewright and Farquhar therefore conducted a systematic search and narrative review of COPD carer support needs and mapped these to the fourteen domains (questions) on CSNAT v2 to determine its comprehensiveness for COPD carers.^3^ The review concluded that, while CSNAT v2 encompassed most COPD carer support needs, the addition of a domain relating to relationship management would enhance comprehensiveness for COPD carers. In parallel, and independently, CSNAT’s developers completed work with carers of patients with Motor Neurone Disease (MND) and came to the same conclusion.^11^ As a result, CSNAT v3 includes an additional evidence-based fifteenth domain relating to relationship management.

This study therefore aimed to investigate the face and content validity of the 15-domain CSNAT v3 (including relationship management) with carers of patients with COPD and explore their views on mechanisms for delivery of the CSNAT Intervention with COPD carers in clinical practice. Being a carer-completed tool, establishing face and content validity is essential in order to recommend CSNAT for use with COPD carers in practice. Face validity could demonstrate acceptability to carers and, as the CSNAT was also designed to be holistic, demonstrating content validity would confirm breadth of coverage and suitability for purpose.^12^ Further, confirmation of face and content validity would enhance practitioner confidence in the tool with COPD carers.

## Methods

### Design

The study design drew on the pragmatist paradigm, utilising the method best suited to the research question: in this case, focus groups.^13^ Focus groups can achieve greater depth of understanding through their unique features enabling retrieval of data that may otherwise be missed: interactions between participants, within-session discussion of diverse viewpoints and experiences, and the greater ease participants can feel in an informal group setting.^14^ Ethical approval was obtained from University of East Anglia’s Faculty of Medicine and Health Sciences Research Ethics Committee (Reference: 201819–101).

### Sampling and recruitment

Participants were recruited via Breathe Easy support groups currently, or previously, affiliated with the British Lung Foundation; the groups provide peer support and education for people living with respiratory conditions. East Anglian group leaders were invited to share study information with their members. Recruitment packs (letter of invitation, participant information leaflet, reply slip and stamped-addressed envelope) were then provided to interested group leaders for distribution to members with COPD, or those who supported someone with COPD (carers). Patients were asked to pass on packs to their carer if this person did not attend the group. If returned reply slips indicated interest, the study researcher (KM) made contact to answer any questions and arrange the focus group for the locality. Following focus group completion, Breathe Easy groups were given a modest donation for facilitating recruitment.

### Data collection

Four focus groups were held (September–October 2019). To enable participation, we used previously employed successful strategies including hiring attractive venues chosen with participant accessibility, comfort and convenience in mind: hotel meeting rooms (n = 3) and a community centre.^15^ Refreshments were provided and travel costs reimbursed. Before the focus group discussion commenced, written informed consent was obtained and participants completed a brief demographics questionnaire. Groups were co-facilitated by both authors, lasted approximately 90 minutes, and were audio-recorded (with permission); fieldnotes documented non-verbal behaviour between participants.

A topic guide ensured key topic coverage. Participants were first given ground rules and reminded that the group’s purpose was data gathering for a research study rather than as a support group (although it was acknowledged that participating could be supportive). The group was first asked what support they received as a carer and from whom. The CSNAT and CSNAT Approach (which comprise the CSNAT Intervention) were then explained to the group, before participants were given printed copies of CSNAT v3 (the tool). Participants were then asked to share initial thoughts on its layout and wording, followed by discussion of each of the 15 CSNAT domains (as listed in Column 1, Table 1[Table table1-1742395321999433]), considering their relevance to COPD carers. Carers were also asked if any areas of support need were missing.

**Table 1. table1-1742395321999433:** Responses to individual CSNAT domains by COPD carers.

CSNATv3 domain	Narrative summary	Supporting quotes
1. Understanding your relative’s illness	Understanding COPD was important for: 1) carers’ own peace of mind, 2) forming expectations for the future and 3) so that they could better care for the patient. Learning about COPD could be difficult: some doctors were not transparent about the diagnosis, seemingly reluctant to diagnose it (being a terminal illness) and communicate its implications clearly to the carer (which carers felt would have been useful). Carers sometimes struggled to take in the information at diagnosis but would have found discussion of next steps helpful at some point. Sometimes it was difficult for carers to ask more about the condition in front of the patient. Carers also struggled with ‘COPD’ being a very general term.	*‘… as I’ve said for years and years I didn’t really understand it [COPD]…they [healthcare professionals] were almost unwilling to name it and…it's very difficult if you've got a partner who at that time was really compos mentis and understood everything, for me to ask any questions.’* (Joanne, FG1)*• ’If I know the condition better I can help her [the patient]…I don’t think anybody’s actually ever sat down and said, “Well your partner’s got COPD, this is how it’s going to affect them.”* (Roger, FG3) *• ‘And also sometimes the doctors don’t explain everything, do they?…And you can look online…and it scares you to death.’* (Amy, FG4)
2. Having time for yourself in the day	Carers thought this domain relevant even if not currently a problem for them. All felt that having time for themselves was important for their wellbeing, no matter how good their relationship with the patient. One carer felt that although she technically received respite she used that time to do chores. Another had learnt to compromise by lowering her standards for household maintenance in exchange for having more personal time as they felt this more “valuable”. Some felt the patient would be unable to cope if they left them to take time for themselves, or that even if they had time away they would not have the energy to do anything. Others talked about being able to go out but never having time alone at home due to the constant presence of the patient.	*‘I mean, I can go out. But you don’t get time in your own home. That’s the difference.’* (Imogen, FG2) • *‘…he [the patient] says that he won’t go anywhere without me.’* (Jeanette, FG2) • *‘No matter how much you love somebody you need a bit of time to yourself.’* (Roger, FG3)
3. Managing your relative’s symptoms	Carers agreed there was a lot to learn about managing symptoms, including interpreting phlegm colour and use of medications. They described difficulties including: getting incorrect, incomplete or inconsistent information on medication administration from healthcare professionals; struggling to monitor patient compliance with medication regimes; and the patient or carer finding medication confusing (especially if there was a lot of medication, when tablets looked the same or the packaging changed). Some carers had had to develop their own strategies for managing medication including oxygen equipment.	*‘…he [the patient] was doing all his trays just himself…Half of them [the tablets] were going on the floor, and then we weren't sure if he'd got them in exactly the right days…I was loathe to take over and he's not someone who likes to be taken over.’* (Joanne, FG1) • *‘– he’ll [the patient] take this and he’ll take that and… “What do I take that blue one for? What do I take that yellow one for?”’* (Amy, FG3) • *‘…it* *just came to light that when that does happen and he [the patient] has to have another antibiotic…he should have stopped taking the Colomycin…Well how am I to know that, you know, unless somebody says to you, “Stop taking it”?’* (Jill, FG4)
4. Your financial, legal or work issues	Carers worried about finances including: concerns about taking on responsibility for managing financial issues, diminishing retirement savings, affording equipment/home adaptations/assistance with household tasks now beyond both patient and carer, unclear advice on eligibility for financial support, and not knowing where to get advice. They discussed issues such as power of attorney (some had completed these arrangements) and varying eligibility status for Carer’s Allowance. Sources of information varied: some had independently explored benefits options while others had received information via Breathe Easy.^a^ Several felt support for working carers was particularly important, including one carer who had given up paid work to care full time.	*‘Paying the water bill, the electricity, he [the patient] knows when it's all due…you give the reigns over to somebody else and you've got out of the habit and I think, "I must pay more attention". But then because he just automatically does it…’* (Kate, FG1) • *‘We struggle because that money may not seem a lot to anybody… you know, we’re on a fixed pension so we can’t work an extra weekend to pay for it, we’re on a fixed amount.’* (Phyllis, FG4) • *‘…we’ve received quite a bit of financial information through the Breathe Easy group, benefit people, etc, coming and talking and just telling us who to contact…unless you know where to go to get help…I think these people that have got nobody in their corner, I just don’t know how they manage with it all.’* (Jill, FG4)
5. Providing personal care for your relative	Several carers helped with personal care, including: helping with getting in or out the bath, hair washing, dressing and managing double incontinence. Their assistance was often needed: patients found steam or bending down triggered breathlessness. Carers wanted to pre-emptively learn more about aids and equipment to help with these tasks even where patients were not yet needing much help with personal care; it was an important consideration for the future which carers had clearly thought about.	*‘It might be helpful to know of some of the aids you can [get] – stools and bathing boards and things like that. I mean, you find out about those things gradually but if you learnt about them a bit earlier it might have been useful.’* (Roger, FG3) • *‘…at the moment it’s only now and then that he [the patient] does need help like that. Yes, in the future it will be permanent, yeah, so I think it’s [CSNAT domain] a very good thing to add.’* (Phyllis, FG4)
6. Dealing with your feelings and worries	The impact of caring on emotional wellbeing was frequently discussed. There were worries and frustrations including: the unpredictability of COPD, managing COPD and co-morbidities, accessing services, and their role as proxy for the patient. A concern that emerged across focus groups related to how both patient and carer could struggle if the carer became ill or injured – one carer had felt “lucky” to have contracted the flu while the patient was in hospital and supported. Carers discussed how they had become accustomed to worries, but some appeared resigned to their strategies for managing them rather than satisfied with these. One carer stated that worry was what tired her the most and another stated that this domain would be *“really quite high”* in terms of its importance to COPD carers.	*‘…that’s my main concern, is if anything happens to me; I don’t know quite how we would manage.’* (Imogen, FG2) • *‘And I think with hospital visits, beside it being stressful in itself, can make us feel we’ve lost control because all of a sudden it’s in someone else’s hands and “Do they know exactly what my husband wants?”’* (Phyllis, FG4) • *‘And when you're very tired and something happens, you know, if we have a real problem with a bowel movement when I'm just about to go to bed or something… I'm exhausted and I just don't want to deal with this…over time I've found the way to deal with it is just to say, well, that's what it is.’* (Joanne, FG1)
7. Managing relationships	Despite not identifying this domain as a new addition to the CSNAT for v3, it elicited strong reactions. Some carers were immediately emphatic about its importance; others were initially dismissive. Interestingly, even when carers questioned this being on the CSNAT, almost all went on to discuss issues that would suggest its relevance. Relationships with the patient had changed over time. Tension was a frequent experience. Patients struggled to reconcile their wish to remain independent with the reality of needing help from the carer, causing frustration for both. Several carers spoke of how they missed completing certain activities with the patient. Carers sometimes seemed disappointed by limitations imposed by the patient’s health: several mentioned a desire to go abroad or to special events but feeling unable to leave or travel with the patient. Two carers talked about how changes to the patient’s cognition (due to a comorbidity) had caused their relationships to change. Carers also spoke about relationships with other family members including both positive, supportive relationships and problematic, unsupportive relationships - unhelpful family sometimes in denial of the patient’s declining health.	• *‘We [carer and patient] have a very good relationship but it [COPD] shapes it in some ways.’* (Roger, FG3) • *‘Well I think it changes your relationship with the person that you’re caring for… there are certain things that you can’t do together…they get very frustrated as to what they can’t do compared to what they used to be able to do and I think that has a knock-on effect…they might be a bit snappy, they might not be as cheerful as they used to be, simply because they’re ill and they can’t do what they used to be able to do.’* (Imogen, FG2) • *‘… I* *can’t cope with it…we’ve always had such a good marriage and a very adventuresome marriage, there’s so much I want to do and I can’t…everything has to be by car, because he can’t go on a train because he can’t walk – and you think, “When do I start my life?” because those are the things that I’d like to do.’* (Phyllis, FG4)
8. Knowing who to contact if you are concerned about your relative	Many did not know who they would contact for help and advice. Carers mentioned a range of services but were not always clear which service was currently responsible for the patient’s medical care. All lacked confidence in NHS 111.^b^ Several wanted access to someone familiar with their situation that they could ring for advice, including at night when most crises seemed to happen.	*‘You wish there was somebody on the end of a phone all the while. You know, that you can just ring up and ask the questions…the same person, the person who knows you.’* (Lillian, FG1) • *‘No, I’m not ringing 111. I have and you get an absolute load of rubbish – they don’t know what they’re talking about, with all due respect. I’ve just put [the patient] in the car and taken her to [local hospital].’* (Roger, FG3) • *‘I* *don’t know anybody…so that’s a very, very important one [CSNAT domain], yes.’* (Phyllis, FG4)
9. Looking after your own health	Maintaining their own health was seen as vital. There was concern about becoming ill or injured and the effect this would have on the ability of both carer and patient to manage. One carer had reluctantly moved into a separate bedroom for more rest, as she was starting to forget her own medication and health needs. Carers often had their own health conditions which were not always obvious to others but affected their ability to manage day-to-day.	*‘…you do the person no good; if you can’t look after yourself you can’t care for them, can you, you know?’* (Amy, FG3) • *‘And I think that is probably what worries us all in the event…for instance, we have an accident and break our arm, we can’t drive, you know, those sort of things.’* (Jill, FG4) • *‘Yeah so, looking after your own health certainly. And you - especially I found it difficult because people who know me don't see anything wrong with me…But there are things wrong with me that sometimes impinge on what I'm doing and that's not always visible.’* (Joanne, FG1)
10. Equipment to care for your relative	Carers talked about a range of equipment that they had either purchased, rented or received via NHS services, some of which was highly valued e.g. one carer spoke about how a scooter had enabled her and the patient to have ‘more of a life’. Carers discussed negative experiences trying to obtain appropriate equipment including difficulties with NHS equipment provision due to living on a county border. They worried about the financial ramifications of buying or renting equipment.	*‘…there was support with things like stairlifts and wet rooms and things like that but I understand most of that has disappeared. I don’t know if it has or has not, you know?’* (Roger, FG3) • *‘[Regarding equipment hire:] It’s knowing where to go without being ripped off by someone, you know, “Oh here’s someone who’s vulnerable,” and knowing where to go and get the right thing.’* (Phyllis, FG4) • *‘Occupational therapy are not easy to deal with.’* (Chelsea, FG2)
11. Your beliefs or spiritual concerns	Not all carers felt this domain personally relevant but could see relevance for others. Carers talked about their varying engagement with spirituality and support from religious groups, and the likely difficulties for carers who were unable to attend to spiritual matters as before. They spoke in broader terms, questioning why the patient had become ill and why this had happened to them, but also reflected that doing so ultimately didn’t help. When discussing this domain, one carer talked about how he and his partner disagreed about him planning to donate his organs.	*‘[Regarding church attendance:] Yeah, people might be concerned that, you know, “I’ve always done this and now I can’t” and it pulls you.’* (Chelsea, FG2) • *‘I think in the early days I questioned, “Why […]?” but it doesn’t do you any good thinking about it.’* (Roger, FG3) • *‘Yes, I think that wouldn’t be for me but for somebody who is very spiritual then I think that [domain] would be very important… And I think for that to be addressed would give them a lot of comfort.’* (Jill, FG4)
12. Talking with your relative about his or her illness	Some carers felt it was easy to talk to the patient about their health but could see how others might find this more difficult if the patient was a private person, felt negatively about their condition or was not accepting of it. One carer interpreted the domain in relation to motivating her husband to engage with exercise, which she found difficult at times because she felt she had to “bully” him. Some linked this domain to talking to other relatives rather than the patient e.g. one carer spoke of struggling to talk about her husband’s condition with their son.	*‘And I’d love to know how to tell him [son], you know, “This is exactly what he [the patient] has gone through.” I mean, I explained to him [son]…and I just felt it went…[Whooshing noise].’* (Phyllis, FG4) • *‘We do [discuss COPD] all the time […] but I think for somebody who is in the position where they’re negative about it or not accepting it then it must be very hard.’* (Jill, FG4) • *‘I can understand people wouldn’t…you know, they’re quite private and they wouldn’t want others to know.’* (Amy, FG3)
13. Practical around the home and elsewhere	Carers talked about external help they had to manage household tasks (often with cost implications) or strategies used to complete tasks. They talked about increasingly struggling to have a break from household tasks, particularly as the patient became less able to help. They also spoke about how the patient could struggle to accept that they were no longer able to contribute as much towards household tasks and how this could be a barrier to the carer getting access to external help. Others were aware that external help might be needed in the future.	*‘I think it’s because you have to do it on your own; when you do it together it’s a pleasure but knowing you’ve got to do it on your own, it becomes a chore.’* (Phyllis, FG4) • *‘…it seems silly to say, you're doing everything. You're washing the car, you're filling the car with petrol, you're cooking the dinner, you're walking the dog. And there's nothing, there's no break from it, there's no…there's nothing that he [the patient] can do…’* (Joanne, FG1) • *‘…and then these jobs used to pile up because if I said anything about “Ooh, we’ll get somebody in,” [the patient would respond:] “Oh I can do that.”…[…] he doesn’t like to think that he can’t do things’* (Mabel, FG4)
14. Knowing what to expect in the future when caring for your relative	Carers interpreted this domain in different ways, speaking about the future in terms of 1) financial and legal concerns, 2) anticipated disease progression, and 3) funeral planning. Some described how they had been preparing for the future (e.g. arranging Power of Attorney), whereas others chose not to. Referencing the unpredictable nature of COPD, they felt that learning more about the disease and available support could be helpful and discussed how unpredictability led to living ‘day-by-day’. The variation in carers’ willingness and desire to talk about this topic with the patient was notable, including one carer stating they wanted to but did not know how to broach the topic. Even when carers did not want to discuss the future with the patient, they could see the domain’s relevance.	*‘And if someone says something is sort of life-limiting you think, “Oh they’re going to die tomorrow,” you know, as well, you know, that’s sort of like the fear, isn’t it, really of the long term.’* (Amy, FG3) • *‘“Knowledge is power” […] It’s not necessarily you’re going to be doing it but at least you know in your mind that if something happens that’s the road you’d go down.’* (Jill, FG4)
15. Getting a break from caring overnight	Carers discussed being vigilant overnight. Some said they did not currently need support with this but felt it could be very important for certain carers as sleep was vital to daytime functioning. Others did need support overnight, but would not know where to go for it, or how to manage guilt at leaving the patient overnight. They often found the patient’s oxygen equipment disrupted their sleep.	*‘And when the NIPPY’s on too… Some nights it bothers me…But I don’t think I’d want to be away from him [the patient] at night really because you worry about them too much’* (Jeanette, FG2) • *‘Overnight is not really an issue yet but if one of us gets a lot worse it might be.’* (Roger, FG3) • *‘… I think it’s very important because you need your sleep, you know, you need for you to be able to get through the day and function, and so it could be very, very important for people.’* (Jill, FG4)

^a^British Lung Foundation peer support groups.

^b^NHS service that provides advice and referrals via telephone or the internet for urgent (but non-life-threatening) medical issues.

Discussion then moved to delivery of the CSNAT Intervention in clinical practice. This explored carers’ views on (1) the most appropriate individuals to deliver the intervention, (2) appropriate settings for discussions around support needs identified by CSNAT, and (3) how these might be followed up, including when the CSNAT should be completed again.

At focus group closure participants were thanked and advised to contact the research team (both registered health professionals) if they needed support resulting from participation.

### Data analysis

Audio-recordings were professionally transcribed. The study researcher (KM) checked and anonymised transcripts against the audio-recordings (allocating participant pseudonyms), whilst enhancing data familiarity. Interviewee transcript review (ITR) was not conducted in order to accurately preserve what was said; ITR can sometimes lead to alteration or removal of relevant data.^16^

A narrative analytic approach was taken, utilising Framework Analysis.^17^ Focus group discussions were largely structured around the 15 CSNAT v3 domains. The domains thus provided the initial framework which was then added to as analysis progressed, enabling identification of consistencies and divergences in thoughts and experiences across participants.^18^ Coding was completed in NVivo 12 Pro, then charted into a framework matrix in Excel by KM. Regular research team meetings involving iterative re-examination of transcripts ensured participants’ voices were retained whilst successfully distilling data consistently into appropriate codes to enable development of emerging themes.

### Patient and Public Involvement (PPI)

A Carer Advisory Group (CAG) consisting of bereaved and current COPD carers provided PPI (2 sessions). The CAG considered the acceptability and comprehensiveness of topic guide questions, ways to sensitively approach topics, resonance of the findings with their own experiences and the appropriateness of the researchers’ interpretations.

## Results

In total, 62 recruitment packs were provided to group leaders, with a further four groups opting to send digital versions to members via email. The denominator is unknown therefore a response rate cannot be calculated. Thirteen carers responded: one was ineligible as the person they supported did not have COPD, while another declined participation stating that caring had not impacted on them significantly (although this was not a requirement). Eleven COPD carers were successfully recruited and participated: most were female (n = 10), their ages ranged 52–79 years. COPD patients (*n* = 2) also attended one group to facilitate attendance of their carer: one was their carer’s husband while the other was their carer’s mother.

### Current support

Carers described support received or helpful services they had been signposted to. Helpful inputs included practitioners that took an interest in them, provided reablement care or equipment, or referred them to useful services (e.g. assistive technology or counselling sessions). However, this was not always the case: *‘… your partner is getting the best care and attention but at the end of the day, whether they’re at home or in hospital, it is the partner, the carer that has just been, I feel, abandoned…’* (Phyllis, FG4).

Some carers were supported by their wider family. Carers also mentioned supportive organisations such as Breathe Easy and Carers Matter Norfolk (regional carer services hub): *‘…they’ve been extremely helpful in helping me to plan a way forward…’* (Joanne, FG1). However, others reported having no support and several felt unsupported even if they went on to mention input that might be considered supportive: *‘I’ve been given lots of numbers and associations to go to but it’s very hard, it’s very slow, everything you either have to fight for or wait, so there is nothing there.’* (Phyllis, FG4)

### Experiences of caring

COPD was challenging due to both its unpredictability and because patients often looked well, belying difficulties: *‘And then he sits in the restaurant and somebody would come in and they’d say, “Ooh you look well”. And I’m thinking, “Do you know the effort to get us here today?”’* (Mabel, FG4). Carers described life as less “spontaneous” because of COPD. Caring became more challenging over time as COPD (and comorbidities) progressed. Carers described the difficult balance between providing support and being considered overbearing: *‘But I do try to say nothing because they must think “Oh, here’s that busybody wife again”.’* (Amy, FG3). Most had few breaks from caring, compounded by patients being able to offer gradually less help with household tasks.

Experiences of health and social care were mixed, with discussions largely negative. Carers spoke of staff that were knowledgeable, helpful and understanding of their situation, but when these staff moved on, or services suddenly ceased, adjustment was difficult: *‘…my husband was under a super doctor at our surgery and he’s left…so we’ve got the job of, you know, getting to know a new doctor…we’ve got to sort of build up a relationship…’* (Lillian, FG1). Factors negatively affecting patient care (and carers experiences) included: service and equipment access, lack of continuity in care and information, and poor interprofessional communication. Some carers felt healthcare professionals lacked time to talk to them: *‘I mean, you said this is for when a health professional talks to you about caring. When does anybody have-? Nobody’s ever done that.’* (Roger, FG3).

### Face and content validity of CSNAT v3

All carers agreed that CSNAT v3 was easy to understand, the instructions made sense and it was easy to complete. Summaries of their discussions of each of the 15 CSNAT domains are presented in Table 1, with supporting quotes.

None of the CSNAT v3 domains were considered redundant. While carers generally felt that, together, the domains were comprehensive, when asked whether there were additional concerns not covered by the tool two carers referred to travel issues: one mentioned travel insurance while another noted challenges in travelling with someone with COPD related to breathlessness, fatigue and managing medications, including oxygen equipment. Through the group discussion these carers agreed these concerns could be raised under other domains (e.g. relating to managing financial issues or patient symptoms).

### Delivery of the CSNAT intervention

Carers suggested a range of individuals could deliver the CSNAT Intervention including doctors, nurses, social workers, carer charity representatives and occupational health nurses. Suggested settings included GP surgeries, hospices, pulmonary rehabilitation sessions or during home visits. Hospitals were generally considered unsuitable: *‘The hospital’s too big and…is more specific to the illness and the medication and not general wellbeing…they’re there for the patient really rather than the person who’s with them.’* (Imogen, FG2); *‘You’d have to have somebody at the hospital that just helps with carers but then you’d have a queue out the door.’* (Chelsea, FG2). Some suggested that delivery would not necessarily need to be professionally led: *‘But what they would have to do is to be trained enough to understand this and be able to put it into action.’* (Joanne, FG1)

While carers identification of individuals and settings for CSNAT Intervention delivery were diverse, there was greater agreement on qualities carers would value pertaining to delivery such as continuity, rapport and not feeling rushed within CSNAT sessions: ‘*You know, if you’re going to do that, it’s not a five-minute job.’* (Roger, FG3). Good understanding of COPD and holistic appreciation of their situation was preferred. They also felt sessions should be one-on-one, with one carer stating a preference for someone not providing direct care for the patient: *‘I like to think there’s somebody I can go to – not that [Patient] can’t go to, but that [Patient] doesn’t go to.’* (Joanne, FG1). Some carers wanted to discuss topics the patient avoided or found difficult, such as planning for the future and what to expect in terms of disease progression: *‘See, we [Carer and Patient] talk about the future but not as he is now, as he was, you know, it’s almost a sort of closed shop, it doesn’t really exist, and he can still do things.’* (Phyllis, FG4). The carers discussed talking to patients about future disease progression:

Jill: *‘And of all of them that’s the one that you don’t really want to have to talk about.’*

Phyllis: *‘No.’*

Mabel: *‘No, we tend to talk about today or maybe tomorrow but I’m afraid we don’t talk about the other. But I would like to know.’* (FG4)

Carers stated that re-visiting their needs using CSNAT should happen at least six-monthly. They felt the time taken to action a response to any identified need would depend on the specific issue and acknowledged professionals’ time constraints. However, all carers wanted to be given a reasonable expectation of when things would be actioned or followed up:

Roger: *‘You don’t mind waiting a bit longer if you know from the outset that it’s-’*

Amy: *‘If it is going to happen, yes.’* (FG3)

## Discussion

This paper primarily reports the face validity of CSNAT v3 with COPD carers. Confirmed face validity is important to reassure practitioners that the tool is acceptable to COPD carers.^19^ The findings also indicate content validity; no domains were superfluous and carers identified no missing support needs. Criterion validity was not assessed; previous validation work with CSNAT v1 suggests this is good.^20^ CSNAT is not a psychometric measure, hence investigation of construct validity and reliability were not appropriate.

CSNAT v3 domains were interpreted in different ways by COPD carers, as evidenced by heterogenous responses to domains such as ‘Talking to your relative about his or her illness’. Responses included 1) speaking to the patient about COPD, 2) speaking to other relatives (e.g. adult children) about patient deterioration and 3) challenges encouraging patient exercise. CSNAT’s developers emphasise that carer-selected domains on the CSNAT are conversation openers and needs subsequently identified and discussed may not always obviously relate to selected domains. As such, domains initially selected are less important than the outcome of the carer-facilitator conversation.^21^

CSNAT v3 included the additional relationship management domain. Although no emphasis was placed on this domain, nor mention made that it was a recent addition, reactions to it were strong with most indicating high relevance. The few carers who were initially less certain of its relevance went on to discuss numerous familial tensions (including patient-carer relationships), thereby endorsing the domain. Further support comes from literature on carers of people with long-term conditions suggesting that managing relationships is an important, yet challenging, aspect of caring in which carers are rarely supported.^22–24^ The data also incidentally suggested relevance of this domain to carers of patients with cognitive impairments: at one group two carers discussed how patients’ comorbid dementia led to patient-carer relationship difficulties. The impact of dementia on relationships is well documented, with subsequent impact on carers’ physical and mental health.^25,26^

COPD carers’ views regarding CSNAT Intervention delivery are also presented. While considerable variation was found regarding carer preferences for where the intervention should be delivered and by whom, they were consistent in describing qualities relating to delivery that would facilitate sessions. This may reflect heterogeneity in services and practitioners carers interact with; they were more likely to suggest practitioners they had positive impressions of regardless of role. Some carers also suggested that CSNAT Intervention facilitators would not necessarily need to be professionals. Together, these findings suggest three implications: (1) that facilitators’ relational skills, demeanour, knowledge and personal qualities are more important than being a specific practitioner type, (2) this element could be incorporated into CSNAT training, and (3) embedding the CSNAT Intervention flexibly into services with varying structures may be possible. However one carer suggested that, due to the busy nature of acute hospitals, staff dedicated to addressing carer support might help: a concept previously mooted.^27^

Some carers preferred the idea of one-on-one CSNAT Intervention delivery sessions (as opposed to jointly with the patient). Practitioners “making space” for carers when discussing support needs has been identified in previous CSNAT studies.^28^ Focusing on carers can help both patients and carers acknowledge the carer’s role, legitimising the idea that carers may have their own support needs.^10,28^ Carers may wish to discuss topics the patient would not e.g. our participants identified “the future” and end-of-life issues. Carers stated that while patients may be reluctant to engage with these topics, they remain important for carers; they can struggle to explore these in front of patients. These findings support recent work with carers of patients with Motor Neurone Disease (MND); carer privacy when completing the CSNAT was identified as a key consideration for facilitating CSNAT Intervention delivery with this group.^29^

Interestingly, the two unplanned patient participants provided contrasting perspectives on this. We had initial concerns that patients’ presence in one focus group might compromise candid discussion between carers, but open discussion of difficulties within carer-patient relationships and how patient and carer support needs may differ suggested this was unfounded. Both patients were supportive of the CSNAT Intervention, however, even though they were appreciative of their carers, it appeared that prior discussion of carer needs had not taken place between these carers and patients. Inclusion of patients in the focus groups enabled observation of patient-carer dyad interactions and interactions with other carers. When one carer stated her (co-present) husband never went out without her, other carers within the group strongly encouraged him to engage with their local hospice day service to give the carer a break, prompting the patient to reconsider this. This suggests that, sometimes, patient presence could help highlight previously unaddressed patient-carer issues, prompting joint problem-solving to resolve them.

This was one of several interesting interactions observed from the focus group format. Also noted was information trading between carers at every group e.g. useful organisations’ and services’ contact numbers. This suggests (1) that despite involvement in Breathe Easy and the extra information this may confer, carers still had unmet support needs and (2) the benefits of peer support.

### Strengths and limitations

This study has limitations. Recruitment difficulties have been previously documented regarding COPD carers, with issues relating to individuals not identifying with the ‘carer’ label and difficulties participating due to caring responsibilities (endorsing our enablement of patients’ attendance when requested).^3^ However no new findings were arising by the end of the final focus group, suggesting sufficient carers had been recruited to meet the study aim.

Carer participants, whilst diverse in age, were all of white British ethnicity; all but one had retired or given up work. Almost all were also women: this is not uncommon in carer studies, and reflects lower numbers of male carers in the general population.^3,30^ Future studies should explore the CSNAT Intervention’s utility with working carers, male carers and those from ethnic minorities. Recruitment was also limited to one region due to budgetary restraints, though participants were from localities across it. Using Breathe Easy support groups as the sole recruitment source, whilst pragmatic, may have biased the sample as group members are likely to be more informed on support. However, finding that many participants did not feel supported may suggest that carers within the general population could be even more likely to benefit from the CSNAT Intervention.

Recruitment numbers and localities resulted in four small focus groups, however data collected were rich, with the smaller group sizes enabling sufficient time for all participants to contribute.^31,32^ As the topic guide required consideration of all 15 CSNAT domains, this was particularly advantageous. Interactions between group members further enriched the data and provided novel insights addressing the study’s aims. An added strength was the integration of PPI: the CAG endorsed the study findings, suggesting trustworthiness in terms of data collection and analytic inferences.

### Implications for practice

Previous work by the authors suggests that COPD carers often have unmet support needs threatening both carer wellbeing and their ability to continue providing patient support. This study suggests that the CSNAT Intervention, using CSNAT v3, is acceptable and valid for identifying and addressing COPD carer support needs.
